# Molecular mechanism underlying cardioprotective effect of dehydroepiandrosterone on endoplasmic reticulum stress induced apoptosis in human vascular smooth muscle cells and human umbilical vein endothelial cells

**DOI:** 10.3389/fphar.2025.1496393

**Published:** 2025-01-28

**Authors:** Ye Zhu, Junxiu Wei, Xin Yang, Wei Zhu

**Affiliations:** ^1^ Department of Obstetrics and Gynecology, Peking University People’s Hospital, Beijing, China; ^2^ Department of Reproductive Medicine, Affiliated Hospital of Hebei University, Baoding, China; ^3^ Department of Immunology, Mudanjiang Medical University, Mudanjiang, China

**Keywords:** apoptosis, dehydroepiandrosterone, endoplasmic reticulum stress, human vascular smooth muscle cells, human umbilical vein endothelial cells

## Abstract

**Introduction:**

This study aimed to investigate the underlying mechanisms involved in the cardioprotective effects of dehydroepiandrosterone (DHEA) on endoplasmic reticulum stress (ERS) -mediated apoptosis in human vascular smooth muscle cells (HVSMCs) and human umbilical vein endothelial cells (HUVECs).

**Material and methods:**

Various concentrations of Dithiothreitol (DTT) were used to induce ERS-mediated apoptosis. DHEA was utilized to inhibit the apoptotic effects of DTT, while estrogen receptor (ER) antagonists ICI 182,780 and G15, the androgen receptor (AR) antagonist flutamide and the aromatase inhibitor letrozole were used to identify the receptors activated during DHEA treatment in HVSMCs and HUVECs. Flow cytometry assessed the apoptotic rate, and Western blotting analysis evaluated the expression levels of ERS-related proteins GRP78 and PERK, and the apoptotic protein marker CHOP. Furthermore, the primary receptor signaling pathways were identified using signaling pathway blockers: LY294002 (PI3K blocker), SP600125 (JNK blocker), and U0126 (ERK1/2 blocker).

**Results:**

In the DTT pretreatment group (0.8 mmol/L, for 8 h), the expressions of GRP78 and CHOP were significantly up regulated, indicating that an optimal ERS model was successfully established. Additionally, 10-4 mmol/L DHEA significantly inhibited the DTT-induced upregulation of GRP78 and CHOP. The results also demonstrated that the apoptotic rate in the DTT group was increased, while DHEA significantly reduced this rate. The addition of ER antagonists ICI 182,780 and G15 to HVSMCs reversed the effects of DHEA; however, the aromatase inhibitor letrozole and the AR antagonist flutamide did not reverse this effect. Notably, the use of the JNK inhibitor SP600125, the PI3K inhibitor LY294002, and the ERK1/2 inhibitor U0126 antagonized the protective effects of DHEA, with SP600125 showing the most significant impact on both HVSMCs and HUVECs.

**Conclusion:**

Our study has identified a novel mechanism underlying the cardioprotective effects of DHEA. Specifically, DHEA may mitigate ERS-induced cell apoptosis by activating estrogen receptors ERα, ERβ, and GPER via the activated JNK pathway.

## 1 Introduction

Globally, cardiovascular disease (CVD) remains the leading cause of premature death despite advances in effective and safe prevention strategies (Zhao, 2021). Research indicates that almost four million deaths occur annually due to CVD in China (Li et al., 2020). Studies have elucidated the role of sex hormones, particularly estrogen in mediating the cardio-protective effect observed in premenopausal women compared to age-matched men (Prabakaran et al., 2021; Ueda et al., 2021). The onset of menopause is associated with a 2- to 4- fold increase in CVD risk among women (Zhao et al., 2018). Estrogen replacement therapy has been investigated as a means to mitigate cardiovascular risks; however the effects of this therapy remain highly controversial (Iorga et al., 2017). DHEA is an essential sex steroid precursor hormone that is secreted by the adrenal glands of humans and other primates (Rosato et al., 2022). The main tissues associated with the metabolism of DHEA include ovaries, testes, placenta, prostate, liver, adipose tissue, and the central nervous system (Klinge et al., 2018). In particular, many organizations’ cells contain enzymes necessary to convert DHEA into androgens and/or estrogens, which enables androgen and estrogen-sensitive tissues to regulate the concentration of sex hormones according to local tissue needs (Rosato et al., 2022). The levels of DHEA change during the aging process. In individuals aged 70–80, the DHEA levels in the blood are only 10%–20% of peak levels (Klinge et al., 2018). DHEA is sometimes used as a supplementary treatment to try to alleviate these symptoms (Rabijewski et al., 2020). Although previous studies have revealed an association between low DHEA levels and cardiovascular risk parameters (Teixeira et al., 2020; Zhang et al., 2022), research on the cardiovascular protective effects of DHEA has not reached a consensus (Iorga et al., 2017). There is also limited evidence for a role in the pathophysiology of a number of other conditions, including insulin resistance and type 2 diabetes (Kiersztan et al., 2021), immune disorders (Sahu et al., 2020; Bongiovanni et al., 2022), malignancies (Williams, 2000), and neurological dysfunction (Sahu et al., 2020; Schverer et al., 2018; Strac et al., 2020). These controversial results necessitate that the scientists worldwide remain dedicated to investigating novel mechanisms and signaling pathways activated during cardiovascular protection by DHEA.

The endoplasmic reticulum serves crucial cellular functions including protein synthesis, maturation, folding and trafficking. Disruption of these functions leads to the accumulation of unfolded proteins in the endoplasmic reticulum,resulting in endoplasmic reticulum stress (ERS). Notably, numerous studies have documented the pivotal role of ERS-related apoptosis in the pathogenesis of CVD through various signaling pathways (Zhou et al., 2021; Tang et al., 2019). ERS leads to the activation of three signaling pathways, namely, protein kinase RNA-like ER kinase (PERK), and activating transcription factor-6 (ATF6). ERS triggers the dissociation of the ER chaperone Glucose Regulated Protein 78 (GRP78) from its luminal domain resulting in the over expression of GRP78 and the activation of signaling pathways aimed at restoring endoplasmic reticulum homeostasis (Amen et al., 2019). However, during intense ERS, the signaling pathways that mediate post-translational attenuation are inhibited leading to the further accumulation of unfolded protein aggregates and the initiation of C/EBP homologous protein (CHOP)-mediated apoptotic cell death (Li et al., 2017; Azhary et al., 2019).

Human vascular smooth muscle cells (HVSMCs) are crucial for vascular structure and function, participating in the regulation of vascular contraction and relaxation (Zhang et al., 2019). Their abnormal proliferation and dysfunction are closely associated with CVD (Stojanović et al., 2020). Human umbilical vein endothelial cells (HUVECs) form the inner wall of blood vessels and play a role in regulating vascular permeability, coagulation, and inflammatory response (Krüger-Genge et al., 2019). Endothelial cell dysfunction is also linked to a variety of CVD (Zhang et al., 2021). ERS significantly impacts the cardiovascular system and is related to the onset and progression of multiple cardiovascular conditions, as it can induce cell apoptosis and exacerbate cardiovascular tissue damage (Chen and Zhang, 2023). Previous studies have investigated the effect of estrogen on the alleviation of endoplasmic reticulum stress in diverse cells. Notably, in the context of the cardiovascular system, Chen and Su have demonstrated that estrogen can modulate the endoplasmic reticulum stress response in specific cell types, which may have implications for understanding the overall physiological and pathophysiological processes related to cardiovascular health (Chen et al., 2022; Su et al., 2017). Our current work aims to build upon these findings and further explore the role of related factors, such as DHEA, in this context.

Pharmacological and molecular intervention studies have demonstrated that the cardioprotective effect of DHEA is contingent upon the activation of the phosphatidylinositol 3-kinase (PI3K)/Akt signaling pathway and the mitogen-activated protein kinase (MAPK) pathway (Colín-Val et al., 2021; Liang et al., 2016). Currently, research elucidating the molecular mechanisms by which DHEA exerts its cardioprotective effects is limited and not widely agreed upon. Therefore, further exploration of the cardioprotective effects of DHEA is warranted.

In the present study, we demonstrate the cardioprotective effect of DHEA on ERS mediated apoptosis and investigate the underlying signaling pathway in an *in vitro* ERS model. Our findings provide novel insights into the molecular mechanisms by which DHEA influences cell apoptosis and enhance our understanding of DHEA’s cardiovascular protective effects.

## 2 Materials and methods

### 2.1 Ethics approval statement

Our project has received review and approval from the Ethics Committee of Peking University People’s Hospital.

### 2.2 Chemicals and reagents

DHEA, selective estrogen receptor (ER) antagonist (ICI182,780), letrozole (LE) and inhibitors (LY294002, SP600125, U0126) were procured from Sigma-Aldrich, United States. Tunicamycin (TM) and G15 were sourced from Tocris Bioscience, United States. Dithiothreitol (DTT) was obtained from Amresco, United States. Cell culture media, including Dulbecco’s modified eagle medium (DMEM), fetal bovine serum (FBS), penicillin, streptomycin, and trypsin, were acquired from Gibco, United States. Antibodies for Western blot analysis, including anti-P-PERK and anti-PERK, were purchased from Cell Signaling Technology (CST). Anti-ATF6 antibodies were obtained from Abcam, UK, while anti-CHOP and anti-GRP78 antibodies were sourced from Santa Cruz Biotechnology, United States. The anti-β-actin antibody and protein ladder marker were obtained from Proteintech Group, United States. Additional reagent-grade chemicals were acquired from Beyotime, Applygen, and ZSGB-BIO in China. The Annexin V-FITC/PI cell apoptosis double dye kit was purchased from BD Pharmingen Company, United States. All other reagents are commercially available.

### 2.3 Cell culture

HVSMCs and HUVECs (a gift from Dr. C.-J. S. Edgell, University of North Carolina at Chapel Hill) were cultured in DMEM supplemented with 10% FBS and a mixed solution of 100 μg/mL penicillin-streptomycin at 37°C in a humidified atmosphere containing 5% CO_2_.

### 2.4 Cell proliferation

The cells were seeded into 6-well plates, and the medium was changed every other day until confluence was reached. Once 70%–80% confluence was achieved, the cells were transferred to DMEM supplemented with 1% FBS for 24 h to synchronize cell growth for subsequent experiments.

### 2.5 Treatment

Briefly, HVSMCs and HUVECs were treated with DTT (0.8 mmol/L or 2 mmol/L) with or without a 10^–4^ mmol/L DHEA pretreatment for 3 h (Ziogas et al., 2020). Selected cells were also treated with 10^–4^ mmol/L of ER antagonists ICI 182,780, G-protein-coupled estrogen receptor (GPER) antagonist G15, AR antagonist flutamide or 10^–5^ mmol/L aromatase inhibitor letrozole. Alternatively, cells were exposed to 10^–5^ mmol/L of post-receptor signaling pathway inhibitors: LY294002 (PI3K blocker), SP600125 (JNK blocker), and U0126 (ERK1/2 blocker) (Kokai et al., 2022).

### 2.6 Western blot analysis

Cells were lysed using a lysis buffer (Beyotime, China), and the cell lysates were boiled for 10 min. The protein content was quantified using the Bradford protein assay reagent (Beyotime, China). A total of 40 μg of proteins was separated by. SDS-PAGE on a 10%–15% gel and then electro-transferred onto a Polyvinylidene fluoride (PVDF) membrane using the Trans-Blot semi-dry electrophoresis transfer cell (Bio-Rad, United States). The blots were rinsed with 1× TBS (Absin, China) containing 0.1% Tween-20 (TBST, Amresco, United States) and subsequently blocked with 5% (w/v) skimmed milk at room temperature for 1 h. Following this the blots were incubated overnight at 4°C with primary antibodies against GRP78, PERK, CHOP (1:1,000), p-PERK (1:500). The membranes were then washed three times with TBST, and incubated at room temperature for 2 h with a horseradish peroxidase (HRP)-conjugated secondary antibody. Immunoreactive bands were visualized using Super Signal West Pico Chemiluminescent Substrate (Thermal Science Pierce, United States). The protein bands were captured and analyzed using a chemiluminescence detector (GE Healthcare, United States). The intensity of the target protein bands was quantified using ImageJ version 1.43 software and normalized to β-actin (1:1000 dilution, Proteintech Group, United States).

### 2.7 Hoechst staining

Cells were seeded into 6-well plates until a seeding density of 30% was achieved. Following a 24-h starvation period, interventions were applied to the cells, after which they were stained according to the outlined protocol. The treated cells were collected and rinsed twice with cold phosphate-buffered saline (PBS). They were then fixed in 4% paraformaldehyde for 10 min, and rinsed again twice with cold PBS. Subsequently, the cells were incubated with Hoechst 33,342 (Sigma, United States) solution at 37 °C in dark for 10 min, followed by two additional rinses with cold PBS (30 s each). Finally, the cells were visualized and images were captured using a fluorescence microscope.

### 2.8 Flow cytometry

Following 24 h of cell starvation, EA. hy926 endothelial cells were treated with DTT and DHEA. Subsequently, the culture medium from the 6-well plates was collected and transferred into 15-mL tubes. The cells were then lysated using 0.25% trypsin (without Ethylenediaminetetraacetic Acid, EDTA) and washed once with cold PBS. This was followed by centrifugation at 1,000 rpm for 4 min at 4°C. After discarding the supernatant, the cell pellet was washed twice more with cold PBS and centrifuged again under the same conditions. The resulting cell pellet was resuspended in 200 μL of 1 × Binding Buffer at a density of 1 × 10^6^ cells/mL.

The cells were subsequently stained with 5 μL of Annexin V and incubated at room temperature for 15 min. Following this, 5 μL of propidium Iodide (PI) was added, and the mixture was incubated in the dark at room temperature for an additional 5 min. Flow cytometry analysis was performed using a BD FACS Calibur (United States). The results were interpreted as follows: normal live cells were negative for both Annexin V-FITC and PI staining (lower left quadrant); early apoptotic cells were positive for Annexin V-FITC staining but negative for PI staining (lower right quadrant); and necrotic or late apoptotic cells were positively stained by both Annexin V-FITC and PI (upper right quadrant).

### 2.9 Statistical analysis

Data are shown as the mean ± standard deviation. Data were obtained from a minimum of five independent experiments. Statistical analyses were conducted using GraphPad Prism 5.0 software. Multi-group comparisons were executed using One-way ANOVA, followed by Tukey’s test for pairwise comparisons. A p-value of less than 0.05 was considered statistically significant.

## 3 Results

### 3.1 DTT-induced ERS mediated apoptosis

**FIGURE 1:**
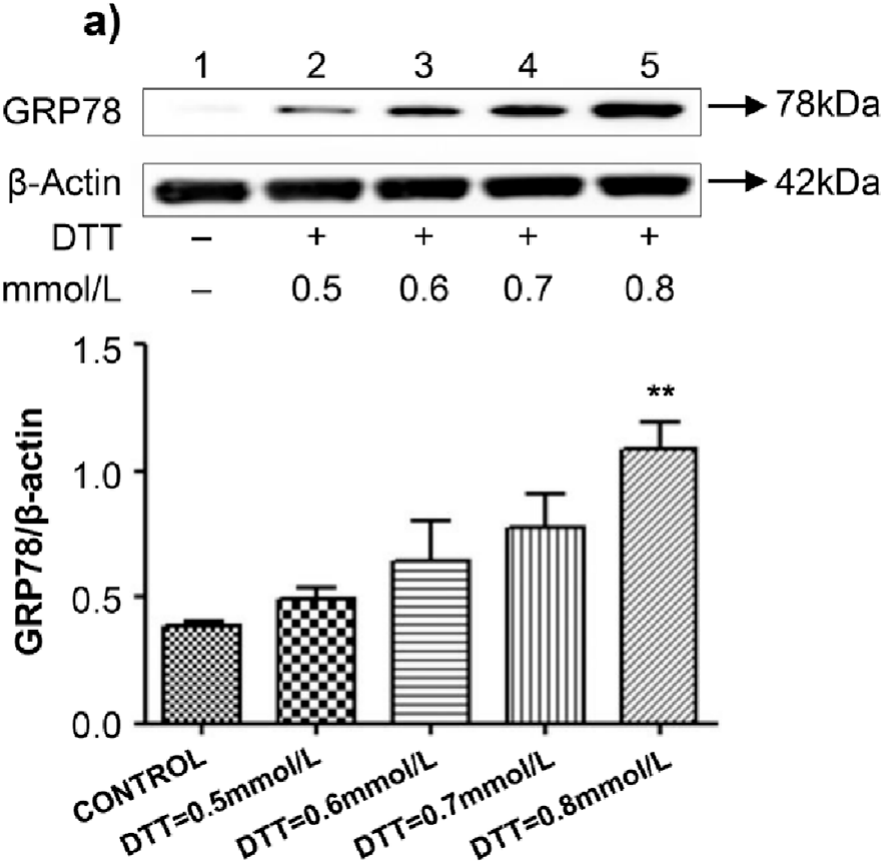
Established ERS model through different conc. of DTT. **(A)** Expression and Quantitative analysis of GRP78 protein in HVSMCs at different concentrations of DTT treatment group. DTT:different concentrations treated for 8 h. N = 5; *P < 0.05, **P < 0.01 vs. Control; #P < 0.05, ##P < 0.01 vs. DTT.

**FIGURE 2:**
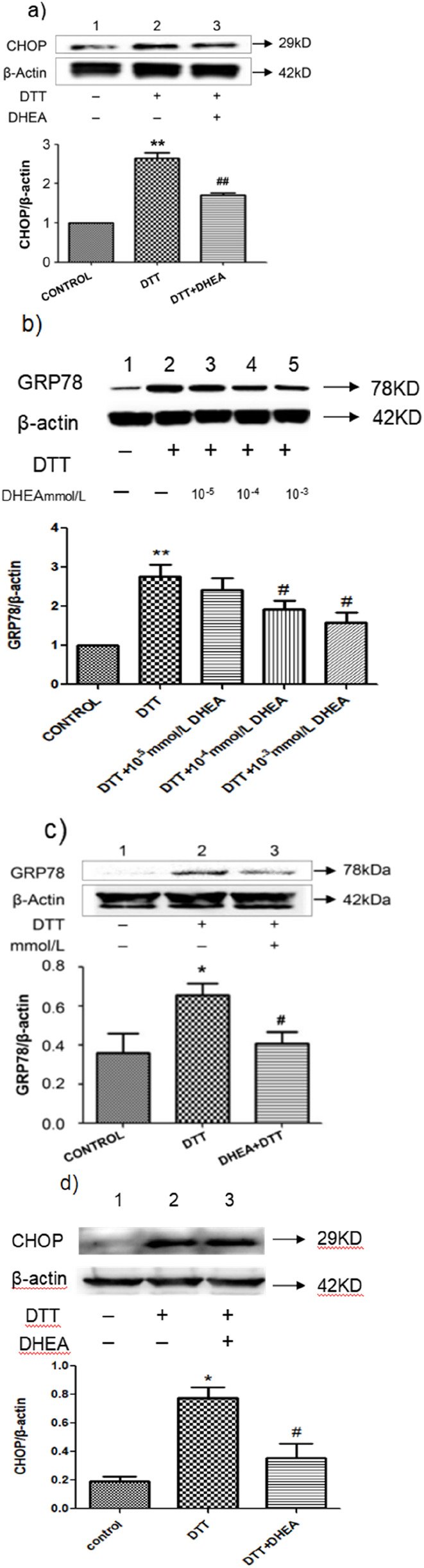
Effect of DHEA on DTT-induced ERS. **(A)** Expression and Quantitative analysis of CHOP protein in HVSMCs after DHEA and DTT pre-treatment. DTT: 0.8 mmol/L DTT treated for 8 h; DHEA: 10^−8^ mol/L pretreated 2 h. **(B)** Expression and quantitative analysis of GRP78 protein in groups treated with varying concentrations of DHEA in HVSMCs. Con, control group; DTT: 0.8 mmol/L DTT pretreatment for 8 h; DHEA + DTT: (10^−5^–10^–3^) mmol/L DHEA pretreatment for 3 h, then add 0.8 mmol/L DTT for further treatment 8 h N = 5; *P < 0.05, **P < 0.01 vs. Con; #P < 0.05, ##P < 0.01 vs. DTT. **(C)** Analysis of GRP78 protein expression and quantification in HUVECs treated with DHEA. **(D)** Expression and quantitative analysis of CHOP protein in HUVECs following pretreatment with DHEA and DTT. Con, control group; DTT: 2 mmol/L DTT pretreatment for 8 h; DHEA + DTT: 10^–8^ mol/L DHEA pretreatment for 3 h, then add 2 mmol/L DTT for further treatment 8 h N = 5; *P < 0.05 vs. Con; #P < 0.05 vs. DTT.

HVSMCs were treated with various concentrations of DTT (0.5 mmol/L, 0.6 mmol/L, 0.7 mmol/L, and 0.8 mmol/L) for 8 h. Notably, treatment with the lower doses of DTT did not result in any significant changes in the expression of GRP78. However, the expression of GRP78 was significantly upregulated by 2.7 times (P < 0.01) in the 0.8 mmol/L DTT treatment group compared to the control group, indicating that 0.8 mmol/L is the optimal concentration of DTT for inducing ERS (Figure 1A). Subsequently, HVSMCs were treated with 0.8 mmol/L DTT for 8 h. In comparison to the control group, the expression of the apoptotic protein CHOP increased by 2.6 times (P < 0.01) in the 0.8 mmol/L DTT-treated group (Figure 2A). These results suggest that higher concentrations of DTT significantly stimulate ERS-mediated apoptosis in the cells.

### 3.2 Effect of DHEA on DTT-induced ERS

We investigated the effect of DHEA on DTT induced ERS by measuring the expression of GRP78 in HVSMCs and HUVECs. The DTT-treated group exhibited a significant increase in GRP78 expression compared to the control group (P < 0.05). However, the addition of DHEA resulted in a decrease in GRP78 expression (Figure 2A). Furthermore, we assessed the impact of physiological concentrations of DHEA (10^–3^ mmol/L, 10^–4^ mmol/L, and 10^–5^ mmol/L) on DTT-induced ERS in HVSMCs. At the dose of 10^–3^ mmol/L and 10^–4^ mmol/L DHEA treatment demonstrated a positive dose-dependent response, significantly inhibiting the upregulation of GRP78 compared to the DTT group (P < 0.05) (Figure 2B). These findings suggest that higher concentrations of DHEA could significantly inhibit DTT-induced ERS in HVSMCs.

Similar results were observed in HUVECs, where after DTT treatment, the expression of GRP78 increased by 1.7-fold compared to the control group (P < 0.05) (Figure 2C). After DHEA pretreatment, the expression level of GRP78 decreased by 35.0% compared to the DTT treatment group (P < 0.05). These findings indicate that DHEA can significantly inhibit DTT-induced ERS in HUVECs.

### 3.3 Effect of DHEA on DTT-induced apoptosis

Excessive and prolonged ERS can lead to apoptosis. To investigate the effect of DHEA on apoptosis, we assessed the expression of the apoptosis marker protein CHOP in HUVECs using Western blot analysis. In comparison to the control group, following treatment with a concentration of 2 mmol/L DTT for a duration of 8 h, the expression of CHOP was observed to significant increase. However, DHEA was found to effectively suppress this upregulation. Specifically, CHOP expression in the DTT + DHEA group was reduced by 48.3% relative to the DTT group (P < 0.01) (Figure 2D). Additionally, flow cytometry was employed to evaluate the apoptotic rate induced by DTT in HUVECs. Exposure to 2 mmol/L DTT for 8 h significantly increased the apoptotic rate (P < 0.05 vs. control group); however, DHEA markedly attenuated apoptosis (P < 0.01) in HUVECs.

**FIGURE 3:**
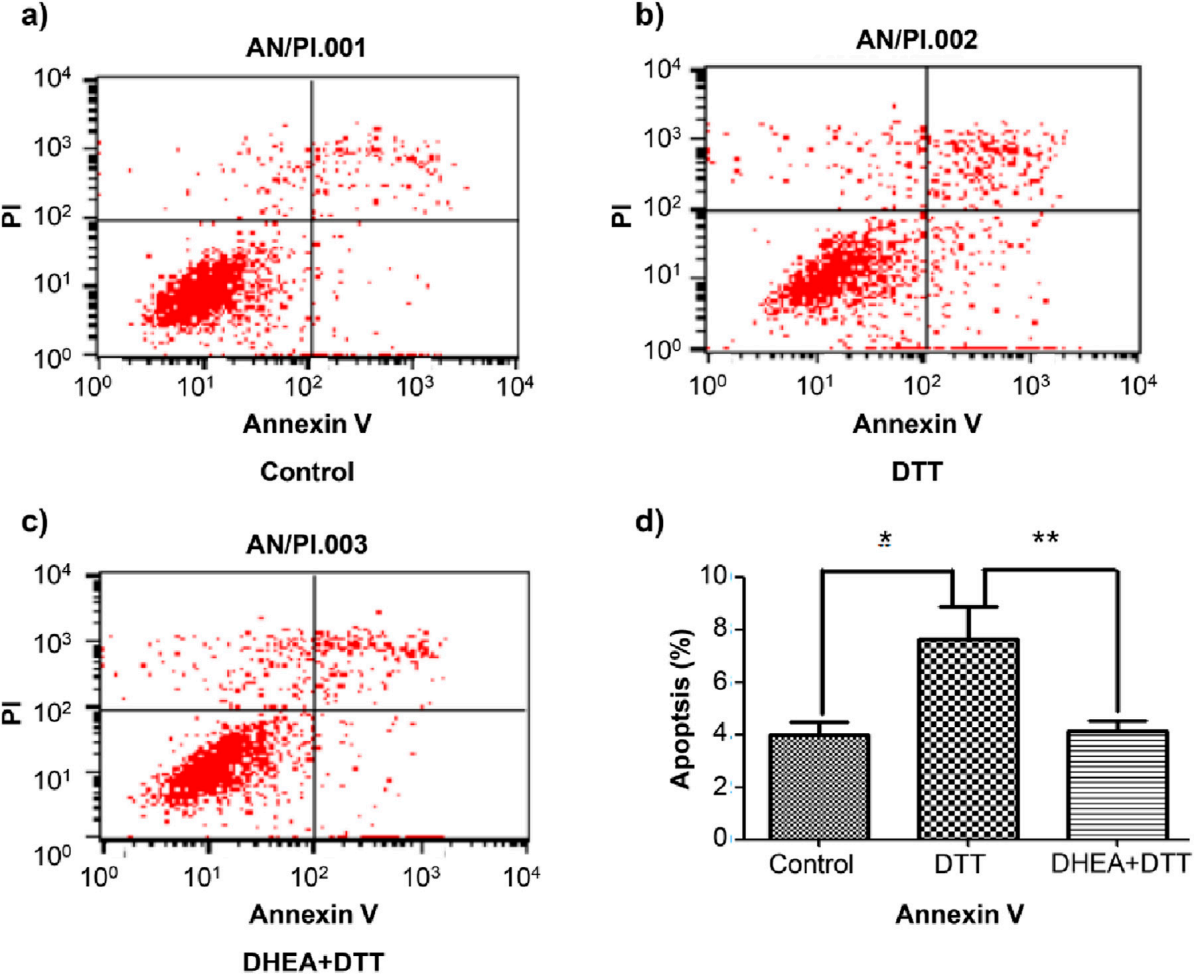
Flow cytometry detection of apoptotic rate in HUVECs. Compared with the control group, the early apoptosis rate increased in the DTT group. After pretreatment with 10^−8^ mol/L DHEA, the apoptosis rate decreased. **(A)**: control group; **(B)**: 2 mmol/L DTT treatment for 8 h group; **(C)**: 10^−8^ mol/L DHEA pretreatment for 3 h, after add 2 mmol/L DTT for 8 h N = 5; *P < 0.01 vs. Con; **P < 0.05 vs. DTT.

Following pretreatment with DHEA at a concentration of 10^−8^mol/L, the apoptotic rate decreased by 42% (Figure 3D), indicating that DHEA protects HUVECs by mitigating DTT-induced cell apoptosis.

**FIGURE 4:**
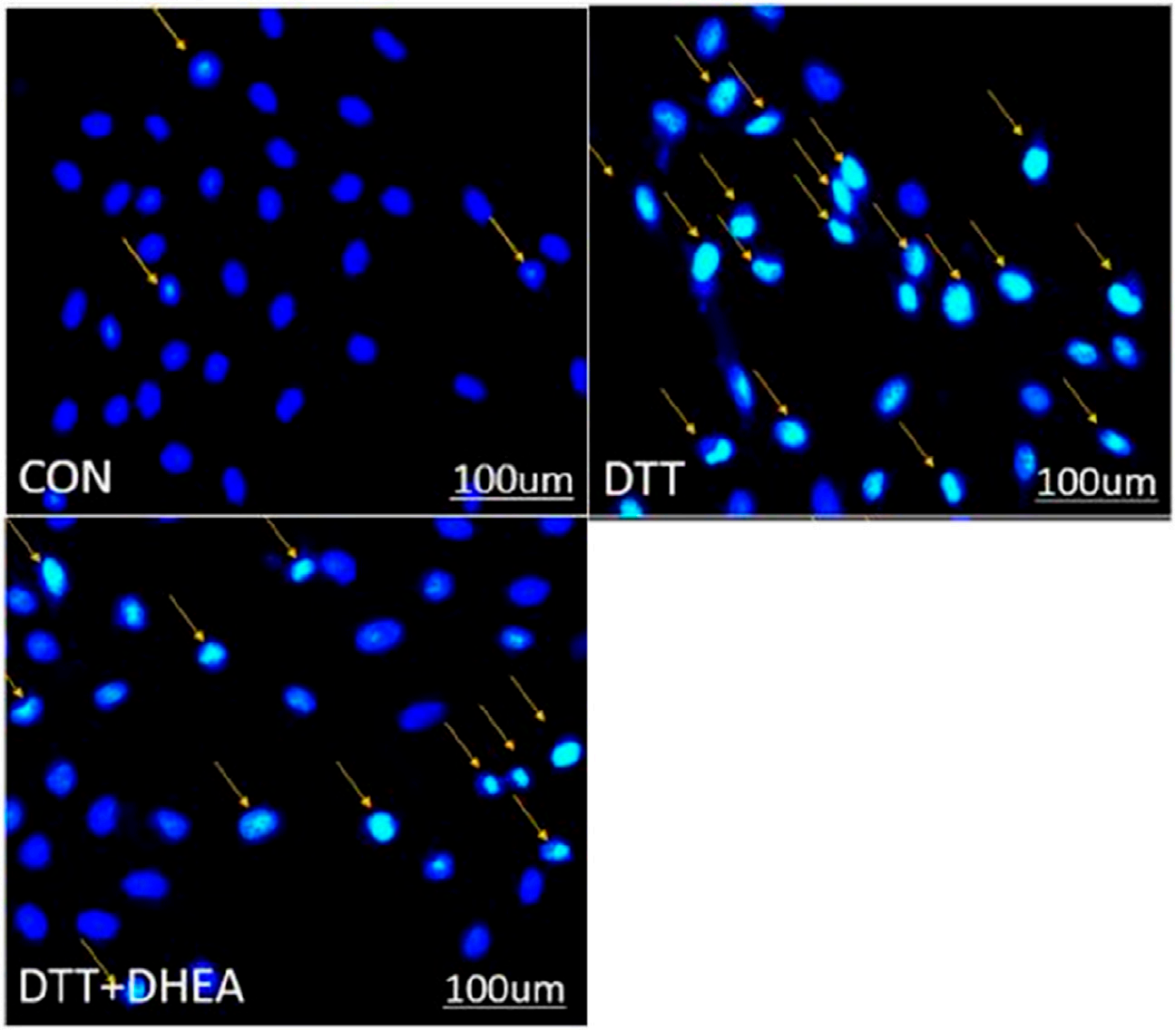
Hoechst staining detects apoptosis in HVSMCs. The orange arrows show the cell nucleus of stained apoptosis. Con: control group; DTT group: 0.8 mmol/L DTT treated for 8 h; DTT + DHEA: 10–4 mol/L DHEA pretreated for 3 h and then 0.8 mmol/L DTT treated for 8 h; N = 3. The excessive endoplasmic reticulum stress may induce the apoptosis while the DHEA could inhibit the occurrence of apoptosis.

Similarly, Hoechst staining demonstrated that the quantity of apoptotic cells in the DTT group was markedly elevated compared to the blank control group within the same high magnification field, and the apoptotic cell count in the DTT + DHEA group was reduced relative to the DTT group. This experiment suggests that DTT triggers apoptosis in HVSMCs by inducing excessive endoplasmic reticulum stress, whereas DHEA exerts a protective effect against cellular apoptosis (Figure 4).

### 3.4 Effect of letrozole on DTT-induced ERS and apoptosis

**FIGURE 5:**
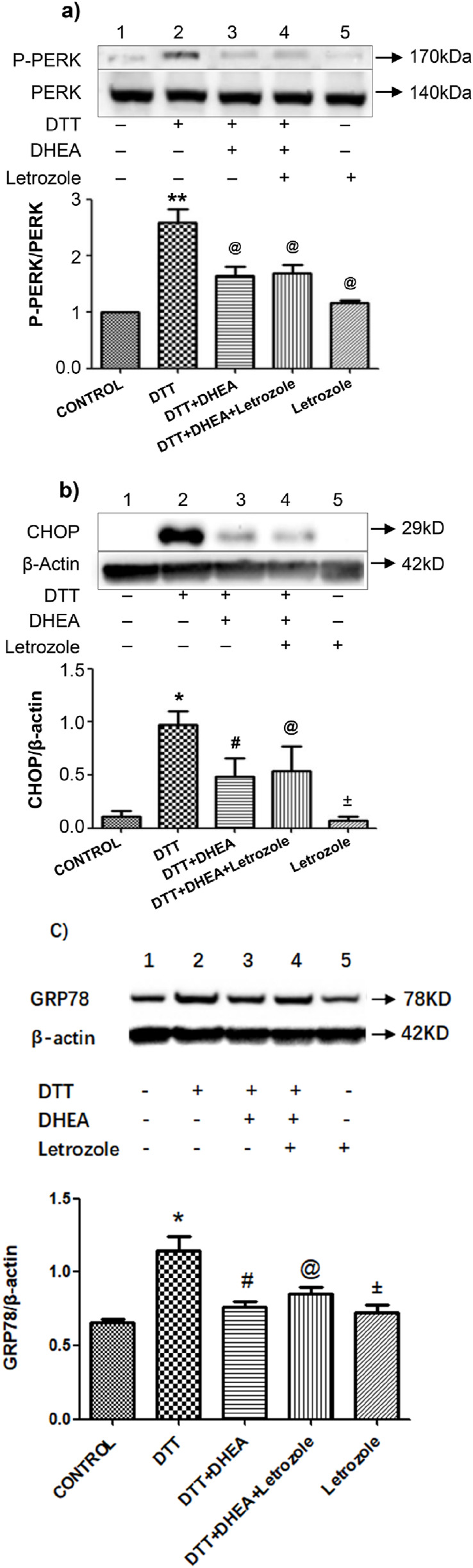
Effects of Letrozole on p-PERK/PERK protein CHOP and GRP78 expression in HVSMCs and HUVECs, respectively. **(A)** Expression and Quantitative analysis of P-PERK/PERK protein expression after DHEA and DTT pretreatment; Con: control group; DTT group: 0.8 mmol/L DTT treatment for 8 h group; DTT + DHEA: 10^−4^ mmol/L DHEA pretreatment for 3 h, after add 0.8 mmol/L DTT for 8 h; DTT + DHEA + Letrozole:10^−5^ mmol/L Letrozole 1 h in advance, 10^−4^ mmol/L DHEA pretreatment for 3 h; after add 0.8 mmol/L DTT for 8 h; Letrozole: 10^−5^ mmol/L. N = 5; **P < 0.01 vs. Con; @P < 0.05 vs. DTT. **(B)** Expression and Quantitative analysis of CHOP/β-actin protein expression after DHEA and DTT pretreatment, Con: control group; DTT group: 2 mmol/L DTT treatment for 8 h group; DTT + DHEA: 10^−8^ mol/L DHEA pretreatment for 2 h, after add 2 mmol/L DTT for 8 h; DTT + DHEA + Letrozole: 10^−8^ mol/L Letrozole 2 h in advance, 10^−8^ mol/L DHEA pretreatment for 2 h; after add 2 mmol/L DTT for 8 h; Letrozole: 10^−8^ mol/L Letrozole. **(C)** Expression and Quantitative analysis of GRP78/β-actin protein expression after DHEA and DTT pretreatment in HUVECs, Con: control group; DTT group: 2 mmol/L DTT treatment for 8 h group; DTT + DHEA: 10^−8^ mol/L DHEA pretreatment for 2 h, after add 2 mmol/L DTT for 8 h; DTT + DHEA + Letrozole: 10^−8^ mol/L Letrozole 2 h in advance, 10^−8^ mol/L DHEA pretreatment for 2 h; after add 2 mmol/L DTT for 8 h; Letrozole: 10^−8^ mol/L Letrozole. N = 5; *P < 0.05 vs. Con, #P < 0.05 vs. DTT, @ P > 0.05 vs. DTT + DHEA, ±P > 0.05 vs. Con.

To elucidate the role of DHEA in DTT-induced ERS-mediated apoptosis, we examined the aromatase pathway. Western blot analysis of p-PERK and total PERK revealed that DHEA significantly inhibited the upregulation of p-PERK/PERK in HVSMCs compared to the DTT group (P < 0.05). Notably, there was no change in the expression of p-PERK/PERK when letrozole was administered in conjunction with DHEA. Furthermore, letrozole alone did not exert a significant effect on the upregulation of p-PERK/PERK when compared to the control group (Figure 5A).

Similarly, the expression of CHOP and GRP78 did not exhibit a significant change upon the addition of letrozole with DHEA when compared to the DHEA + DTT group in HUVECs (Figures 5B, C). Collectively, these findings suggest that DHEA does not inhibit ERS through the aromatase pathway.

### 3.5 Effects of antagonists of estrogen receptors, GPER, and AR on DTT-induced ERS mediated apoptosis

**FIGURE 6:**
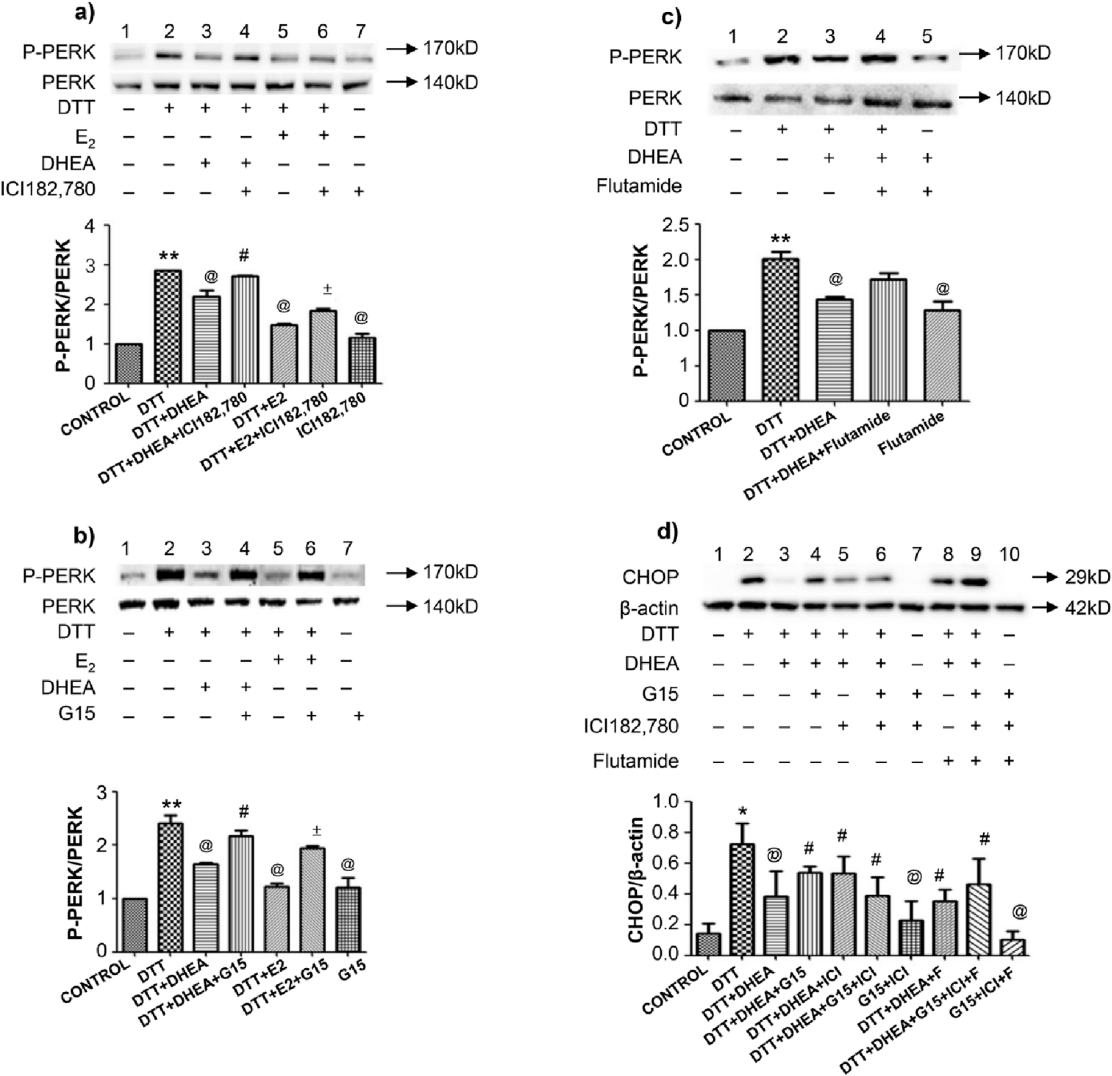
Effects of antagonist of estrogen receptor, GPER and AR on DTT-induced ERS mediated apoptosis on protein expression in various groups. **(A)** Expression and Quantitative analysis of P-PERK/PERK protein after DHEA and E2 along with ICI182,780 and DTT pretreatment in HVSMCs; **(B)** Expression and Quantitative analysis of P-PERK/PERK protein after DHEA and E2 along with G15 and DTT pretreatment in HVSMCs; **(C)** Expression and Quantitative analysis of P-PERK/PERK protein after DHEA along with Flutamide and DTT pretreatment in HVSMCs; DTT + DHEA + G15 or ICI182,780: 10^−4^ mmol/L G15 1 h in advance or 10^−4^ mmol/L ICI182,780 1 h in advance, 10^−4^ mmol/L DHEA pretreatment for 3 h; after add 0.8 mmol/L DTT for 8 h; DTT + E2+G15 or ICI182,780: 10^−4^ mmol/L G15 1 h in advance or 10^−4^ mmol/L ICI182,780 1 h in advance, 10^−4^ mmol/L E2 pretreatment for 1 h; after add 0.8 mmol/L DTT for 8 h; DTT + DHEA + Flutamide: 10^−4^ mmol/L Flutamide 1 h in advance, 10^−4^ mmol/L DHEA pretreatment for 3 h; after add 0.8 mmol/L DTT for 8 h; **(D)** Expression and Quantitative analysis of CHOP protein in HUVECs after DHEA with ICI182,780 G15 or Flutamide respectively and DTT pretreatment. N = 5; *P < 0.05, **P < 0.01 vs. Con; @P < 0.05 vs. DTT; #P < 0.05 vs. DTT + DHEA; ±P < 0.05 vs. DTT + E2.

To validate the role of estrogen and ARs in inhibiting DTT-induced ERS apoptosis, HVSMCs were pretreated with antagonists of estrogen receptors, including ERα (ICI 182,780), ERβ, GPER (G15), and the AR (Flutamide). Western blot analysis of HVSMCs demonstrated that treatment with DHEA and estradiol (E2) significantly (P < 0.05) inhibited the upregulation of p-PERK/PERK compared to the DTT group (Figures 6A, B). However, the protective effects of DHEA and E2 were negated by the addition of ICI 182,780 or G15. Notably, ICI 182,780 and G15 themselves did not exhibit any significant effect on the upregulation of p-PERK/PERK. This indicates that DHEA exerts a protective role against ERS by activating estrogen receptors ERα, ERβ and GPER in HVSMCs (Figures 6A, B).

The AR antagonist flutamide did not inhibit the protective effects of DHEA, as Western blot analysis, revealed no significant changes in the levels of p-PERK/PERK, These results indicate that DHEA does not confer protection against ERS through the activation of ARs (Figure 6C). Furthermore, flutamide itself had no significant effect on the upregulation of p-PERK/PERK.

In HUVECs, the expression of the apoptotic protein CHOP was significantly altered following pretreatment with ICI 182,780, G15 and flutamide. However, ICI 182,780, G15 or flutamide alone did not demonstrate any significant effect on CHOP expression. This finding indicates that DHEA does protect against ERS by activating both ERs and ARs in HUVECs (Figure 6D).

### 3.6 Effects of LY294002, SP600125 and U0126 on DTT-induced ERS and apoptosis

**FIGURE 7:**
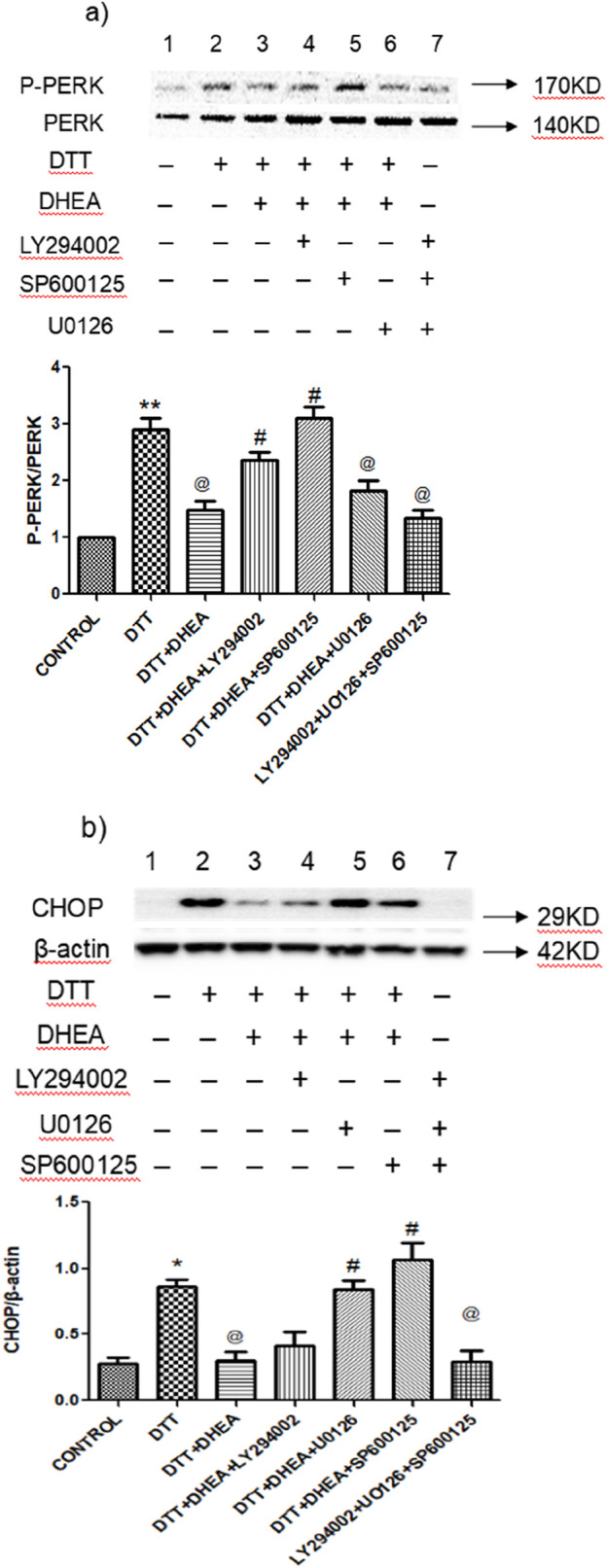
Effects of LY294002, SP600125 and U0126 on DTT-induced ERS and apoptosis on protein expression in various groups. **(A)** Expression and Quantitative analysis of P-PERK/PERK protein expression after DHEA along with LY294002, SP600125, and SP600125 and DTT pretreatment in HVSMCs. **(B)** Expression and Quantitative analysis of protein CHOP/β-actin protein expression after DHEA along with LY294002, SP600125, and SP600125 and DTT pretreatment in HVSMCs DTT + DHEA + LY294002, SP600125 or U0126: 10^−5^ mmol/L LY294002, SP600125 or U0126 1 h in advance, 10^−4^ mmol/L DHEA pretreatment for 3 h; after add 0.8 mmol/L DTT for 8 h; N = 5; *P < 0.05, **P < 0.01 vs. Con; @P < 0.05, @@P < 0.01 vs. DTT; #P < 0.05, ##P < 0.01 vs. DTT + DHEA.

To investigate the post-signaling pathway involved in the inhibition of ERS apoptosis by DHEA in HVSMCs and HUVECs, we assessed the impact of PI3K, C-Jun N-terminal kinase (JNK) and extracellular signal - regulated kinase 1/2 (ERK1/2) inhibitors on the expression of p-PERK/PERK and CHOP in cells pretreated with DHEA. The results demonstrated that DHEA significantly reduced the overexpression of the ERS marker protein p-PERK/PERK (Figure 7A). This inhibitory effect was notably reversed (P < 0.05) upon the addition of LY294002 and SP600125, compared to the DHEA + DTT group. Interestingly, the SP600125 group inhibited a more pronounced inhibitory effect than the LY294002 group.

In HUVECs, DHEA significantly (P < 0.05) suppressed the expression of the apoptotic protein CHOP when compared to the DTT group. Both U0126 and SP600125 were able to negate the inhibitory effect of DHEA on CHOP expression, with the SP600125 group showing particularly promising results (Figure 7B). However, LY294002 did not effectively counteract the effects of DHEA in HUVECs. Notably, the application of LY294002, SP600125, or U0126 alone did not yield significant changes in the expression levels of marker proteins in the ERS model.

These findings suggest that the JNK pathway represents the most promising post-receptor signaling pathway activated by DHEA-activated receptors in HVSMCs and HUVECs (Figure 7).

## 4 Discussion

This study demonstrated that DHEA inhibited ERS induced by DTT in HVSMCs and also reduced apoptosis mediated by ERS in HUVECs. Specifically, DHEA was found to decrease the apoptosis rate in HUVECs by 42%. The protein PERK serves as the primary signaling molecule in the ERS pathway, while CHOP is a key apoptotic marker that is upregulated during ERS (Frakes and Dillin, 2017). Pre-treatment with DHEA led to the inhibition of PERK and CHOP protein upregulation in HVSMCs and HUVECs, respectively. The introduction of ER antagonists ICI 182,780 and G15, to HVSMCs reversed the protective effects of DHEA; however, the aromatase inhibitor letrozole and the AR antagonist flutamide did not alter this effect. In HUVECs, the aforementioned antagonists also failed to reverse the effects of DHEA. Furthermore, the application of JNK inhibitor SP600125, PI3K inhibitor LY294002, and ERK1/2 inhibitor U0126 counteracted the protective effects of DHEA, with SP600125 demonstrating the most significant impact on both HVSMCs and HUVECs. Therefore, it can be concluded that DHEA may exert cardioprotective effects by activating estrogen receptors ERα, ERβ, and GPER through the activated JNK pathway.

When the homeostatic state of the ER is disrupted, a significant accumulation of unfolded proteins occurs, leading to ERS. The unfolded protein response (UPR) is a cellular signaling mechanism that adjusts the ER’s folding capacity by activating stress sensors, namely, PERK and ATF6 to restore protein homeostasis (Madden et al., 2019). Under normal homeostatic conditions, GRP78 binds to each of these sensors, maintaining them in an inactive state. However, during ERS, GRP78 dissociates from these sensors, facilitating the activation of various adaptive signaling pathways (Miyata et al., 2021). The sustained activation of PERK results in the upregulation of CHOP, a transcription factor involved in the regulation of apoptosis (Fang et al., 2017). Furthermore, the expression of the UPR factor CHOP has been shown to be upregulated in ERS models (Miyata et al., 2021).

To confirm the induction of ERS-mediated apoptosis by DTT, we used the specific ERS inducer DTT to induce ERS in HUVEC and HVSMC cells *in vitro* (Fang et al., 2017). DTT disrupts the formation of disulfide bonds (Chen and Zhang, 2023; Ren et al., 2018). This inducer leads to the accumulation of unfolded proteins in the endoplasmic reticulum and induces endoplasmic reticulum stress; therefore, it is used as a classical drug for studying ERS induction. We analyzed the expression levels of GRP78 and CHOP proteins in HVSMCs and HUVECs, respectively. The results of this study indicated that GRP78 expression was significantly upregulated in the group treated with the highest concentration (0.8 mmol/L) of DTT. Additionally, CHOP expression was also upregulated following pre-treatment with this concentration of DTT. Thus, the ERS-induced apoptosis model was successfully established.

HVSMCs and HUVECs are essential components of vascular system, playing a critical role in maintaining normal vascular function. In the pathogenesis of CVDs, such as atherosclerosis (AS), both structural and functional alterations can occur in these cells, resulting in abnormalities that compromise physiological functions (Sun et al., 2020). Research has shown that the functional changes in smooth muscle cells (SMCs) and the abnormalities in the vascular endothelium are associated with several cardiovascular conditions, including AS, hypertension, arterial aneurysms and myocardial infarction (Zhuge et al., 2020). Furthermore, atherosclerotic lesions demonstrate elevated levels of apoptosis in HVSMCs and HUVECs compared to normal vessels (Wu et al., 2020).

DHEA is a steroid hormone synthesized from cholesterol by the adrenal glands (Klinge et al., 2018). In peripheral tissues, DHEA is converted into estrone (E1), testosterone (T), dihydrotestosterone (DHT) and 17β-E2 through the action of aromatase (Tang et al., 2021). The well-documented age-related decline in serum DHEA levels (Zhang et al., 2022) suggests that a relative deficiency of these steroids may be causally linked to the development of various age-associated diseases, including AS. Previous studies have demonstrated that DHEA protects endothelial cells against apoptosis (Kilanczyk et al., 2022; PKiersztan et al., 2017). Interestingly, DHEA exhibits different effects in other types of cells. The results of a study on lung adenocarcinoma A549 and PC9 cells showed that DHEA can leading to the disorder of reactive oxygen species clearance and initiating the apoptosis process of mitochondrial pathway (Zhang et al., 2024). In a study related to polycystic ovary syndrome, researchers discovered that DHEA could suppress the proliferation of ovarian granulosa cells and induce their apoptosis via the PI3K/AKT signaling pathway (Li et al., 2019). To date, there have been few reports on the effects of DHEA mediated by DTT on cardiovascular diseases. In this study, we have shown that DHEA, at physiological concentrations, inhibits DTT-induced ERS- mediated apoptosis in HVSMCs and HUVECs.

Aromatase is the main enzyme involved in the biosynthesis of estrogen in the human body (Ghosh, 2023). Letrozole inhibits the activity of the aromatase enzyme by competitively binding to the heme of cytochrome P450 or CYP450 (Aromatase) (Mukherjee et al., 2022). Accumulating evidence suggests that DHEA exerts vascular effects that are independent of its estrogenic properties (Zhang et al., 2022). Some findings also demonstrate that DHEA and androgens, through genomic and/or non-genomic pathways, have a significant effect on endothelial cell proliferation and migration, vascular contractility, and endothelial pathological processes, such as inflammation, atherosclerosis, and clot formation (Cai et al., 2016). In our findings, we clearly demonstrated that the impact of DHEA on ERS was not inhibited by letrozole in both cells. Notably, anti-apoptotic effect of DHEA in these cell types appeared to be independent of estrogens, as the inhibition of aromatase, the final enzyme involved in the conversion of DHEA to estrogen, did not suppress DHEA’s effects on apoptosis in HVSMCs and HUVECs. Collectively, these findings support the notion that DHEA regulates the function of HVSMCs and HUVECs through mechanisms distinct from those of estradiol.

Previous studies have demonstrated that the activation of estrogen receptors ERα, ERβ, and GPER plays a significant cardioprotective role (Iorga et al., 2017; Aryan et al., 2020; Prossnitz and Barton, 2023). In particular, estrogen receptor ERα is notably effective in protecting against vascular damage (Mahmoodzadeh and Dworatzek, 2019; Fuentes and Silveyra, 2019). Furthermore, GPER activation has been shown to inhibit the proliferation of HVSMCs *in vitro* (Xia et al., 2020; Luo and Liu, 2020). In our study, DHEA was found to reduce apoptosis in both HVSMCs and HUVECs. However, the addition of ER antagonists, ICI182,780 and G15, inhibited the anti-apoptotic effect of DHEA in HVSMCs. This effect could not be abolished by pre-treatment with the ER blockers ICI182,780 or G15 in HUVECs. Thus, it can be inferred that DHEA exerts a protective effect on endothelial cell response by activating estrogen receptors ERα, ERβ, and GPER in HVSMCs.

In this study, we discovered that the action of DHEA could not be completely inhibited by pre-treatment with the AR blocker flutamide, suggesting an AR-independent mechanism in HVSMCs and HUVECs. These results indicate that the anti-apoptotic effect of DHEA is independent of both AR and ER in HUVECs, while it is AR-independent but ER-dependent in HVSMCs. Previous research has reported similar findings, ERs and ARs mediate the effects of DHEA in different ways in various organs (Clark et al., 2018).

Additionally, studies have demonstrated that the p38/MAPK pathway is involved in the upregulation of GRP78 expression during ERS in cells (Zhou and Cai, 2022; Yuan et al., 2021). The PI3K-Akt pathway serves as an anti-apoptotic mechanism and plays a crucial role in cell survival, and proliferation (Deng and Zhou, 2023; Teng et al., 2021). Furthermore, PI3K can directly regulate the MAPK pathway upon activation, including the ERK pathway and the JNK pathways (Guo et al., 2020; Wang et al., 2020; Li et al., 2021).

To investigate the signaling pathways modulated by DHEA, we employed the PI3K inhibitor LY294002, the JNK inhibitor SP600125 and the ERK1/2 inhibitor U0126 in our study. Pre-treatment with each inhibitor mediated the DHEA-induced inhibition of ERS in both HVSMCs and HUVECs, with the JNK inhibitor SP600125 demonstrating the most effect. However, the PI3K inhibitor LY294002 was ineffective in blocking the action of DHEA in HUVECs. Thus, it appears that DHEA may inhibit ERS primarily by activating signaling pathways, with JNK being the most significant. Nonetheless, the precise mechanism by which the JNK/MAPK signaling pathway inhibits ERS warrants investigation.

Our investigation into DHEA revealed significant and innovative findings regarding its ability to attenuate ERS-induced apoptosis. Unlike conventional cardiovascular protective medications or strategies, which typically focused on macroscopic factors such as blood pressure and lipid levels, DHEA employed a fundamentally different approach. It operated from an endocrine perspective, specifically targeting ERS—an essential pathophysiological node in cardiovascular disease. Rigorous experimental evidence convincingly demonstrated that DHEA effectively inhibited the expression of apoptotic proteins and significantly reduced cell apoptosis rates (Lin et al., 2017). This action was mediated through the activation of specific ER receptors and associated signaling pathways, as supported by existing literature. The identification of this novel mechanism not only uncovered new therapeutic targets but also opened new avenues for the development of advanced cardiovascular protective therapies. By investigating intracellular regulatory mechanisms at the microscopic level, our approach offered a promising alternative for patients who had previously shown insufficient responses to traditional treatments, potentially revolutionizing the therapeutic paradigm for cardiovascular diseases.

We conducted a comprehensive comparison of dehydroepiandrosterone (DHEA) and currently available biomedical interventions for cardiovascular disorders. First, we thoroughly described the specific molecular mechanisms by which DHEA exerts its effects. Unlike traditional therapies that often have more generalized or less targeted actions, DHEA precisely targets and regulates the endoplasmic reticulum stress-induced apoptotic pathway. This offers a more direct and potentially more effective approach to addressing the pathophysiological mechanisms underlying cardiovascular diseases. Second, our investigations have provided novel data that highlights DHEA’s enhanced safety profile. In our experimental studies, we observed minimal off-target effects and toxicity, presenting a clear advantage for long-term biomedical applications. This stands in stark contrast to some existing medications, which may cause adverse effects, limiting their use and necessitating close patient monitoring. Moreover, our research into the mechanism of action of DHEA presents new opportunities for the development of personalized and synergistic biomedical strategies.

## 5 Conclusion

Our study has demonstrated a novel approach for understanding the cardioprotective mechanism exerted by DHEA. DHEA ameliorates ERS-mediated cell apoptosis by activating estrogen receptors and utilizing the JNK pathway. These findings provide valuable insights into the action mechanisms of DHEA and its potential use as a therapeutic agent for the treatment of CVD.

## 6 Limitations

In this study, we could have analyzed the expression of additional UPR signaling proteins, specifically ATF6, alongside PERK to provide further evidence for the signal transduction pathways involved in ERS. Additionally, we could have included another group representing a combination of DTT and receptor antagonists to assess the individual effects of the antagonists in the apoptotic cell lines. Furthermore, evidence from *in vivo* studies is necessary to strengthen our findings.

## Data Availability

The raw data supporting the conclusions of this article will be made available by the authors, without undue reservation.

## Glossary

CVD: Cardiovascular Disease
DHEA: Dehydroepiandrosterone
ERS: Endoplasmic Reticulum Stress
PERK: RNA - like ER Kinase
ATF6: Activating Transcription Factor - 6
GRP78: Glucose Regulated Protein 78
CHOP: C/EBP Homologous Protein
HVSMCs: Human Vascular Smooth Muscle Cells
HUVECs: Human Umbilical Vein Endothelial Cells
PI3K: Phosphatidylinositol 3 - kinase
MAPK: Mitogen - activated Protein Kinase
LE: Letrozole
TM: Tunicamycin
DTT: Dithiothreitol
FBS: Fetal Bovine Serum
CST: Cell Signaling Technology
PVDF: Polyvinylidene fluoride
HRP: Horseradish Peroxidase
PBS: Phosphate - buffered Saline
EDTA: Ethylenediaminetetraacetic Acid
PI: Propidium Iodide
AR: Androgen Receptor
JNK: C - Jun N - terminal Kinase
ERK1/2: Extracellular Signal - regulated Kinase 1/2
ER: Estrogen Receptor
GPER: G - protein - coupled Estrogen Receptor
UPR: Unfolded Protein Response
AS: Atherosclerosis
SMCs: Smooth Muscle Cells
E1: Estrone
E2: Estradiol
T: Testosterone
DHT: Dihydrotestosterone
